# Super‐Multiplexed Label‐Free Raman Imaging Uncovers Novel Testicular Metabolic Couplings for Residual Body and Spermatogonial Differentiation

**DOI:** 10.1002/advs.76757

**Published:** 2026-07-21

**Authors:** Jiaxin Li, Xia Chen, Yujuan Qi, Chen Qiao, Qiuru Huang, Dan Zhao, Weiyi Qian, Li Di, Yi Shi, Xiangqi Hu, Yueyue Shao, Kehan Wang, Zhiyu Tang‐Yang, Hanzhi Zang, Yuwen Du, Anran Dong, Jinxiong Feng, Jiayun Chen, Ying Zheng, Xiaofang Tan, Xiaoyu Zhao, Zhonghua Shi, Bo Zheng, Jun Yu

**Affiliations:** ^1^ Clinical Research Center for Reproductive Genetics Center for Reproductive Medicine Affiliated Maternity and Child Health Care Hospital of Nantong University Medical School of Nantong University Nantong China; ^2^ Institute of Reproductive Medicine Medical School Nantong University Nantong China; ^3^ Center for Reproductive Medicine, Affiliated Hospital of Nantong University Medical School of Nantong University Nantong China; ^4^ Department of Reproductive Medicine Xuzhou Central Hospital Xuzhou Central Hospital affiliated to Southeast University Xuzhou Clinical School of Xuzhou Medical University Xuzhou China; ^5^ Department of Clinical Pharmacy Affiliated Hospital of Jiangsu University Jiangsu University Zhenjiang China; ^6^ Reproductive Medicine Center Reproductive Sciences Institute The Fourth Affiliated Hospital of Jiangsu University Jiangsu University Zhenjiang China; ^7^ Changzhou Medical Center Changzhou Maternal and Child Health Care Hospital Nanjing Medical University Changzhou China; ^8^ State Key Laboratory of Reproductive Medicine and Offspring Health Center For Reproduction and Genetics The Affiliated Suzhou Hospital of Nanjing Medical University Suzhou Municipal Hospital Gusu School Nanjing Medical University Suzhou China; ^9^ Department of Histology & Embryology Faculty of Medicine Yangzhou University Yangzhou China; ^10^ SuperVision Medicine Co,.Ltd Wuxi China

**Keywords:** label‐free, metabolic remodeling, raman imaging, residual body, spermatogonial differentiation

## Abstract

The biochemical composition underlying cytoplasmic residual body (RB) and arrested spermatogonia remains unresolved in spermatogenesis, in part due to limited approaches for intact‐tissue metabolic interrogation. Here we develop Super‐multiplexed Label‐free Raman Imaging (SLRI) to generate 2D/3D biochemical maps of intact *Drosophila* testes. SLRI delineates major testicular cell types and provides the first in situ, stage‐resolved mapping of cytoplasmic RBs at two discrete phases. We further identify a differentiation‐arrest‐associated metabolic trajectory in spermatogonial clusters, characterized by coordinated spatiotemporal decay of specific biochemical species. Across 42 spatiotemporally variant metabolites and metabolic pathways, SLRI reveals RB‐ and arrest‐enriched signatures. Notably, RBs and arrested spermatogonia display significant biochemical overlap, alongside unique spectral signatures. Furthermore, we uncovered a ring‐like, punctate arrangement of lipids, amino acids, and vitamins encircling RBs, suggesting structured metabolite partitioning during cytoplasmic remodeling. Collectively, SLRI establishes a general framework for high‐dimensional metabolic mapping and remodeling analysis in intact tissues.

## Graphical abstract

Super‐multiplexed Label‐free Raman Imaging (SLRI) enables 2D/3D metabolic mapping of intact Drosophila testes. Moving beyond descriptive morphology, it establishes a multidimensional tool for tissue metabolic remodeling, and offers a generalizable platform for complex tissue analysis, with implications extending to development and disease.

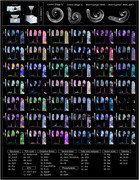

## 1 Introduction

Spermatogenesis represents a meticulously controlled and complex cellular differentiation process that is evolutionarily conserved among diverse species [1]. Although the fundamental events in spermatogenesis are well‐characterized [2], the essential metabolic pathways governing spermatogonial differentiation and the precise biochemical orchestration of cytoplasmic remodeling—particularly during the formation and clearance of residual body (RB) in spermiogenesis—remain inadequately elucidated. These procedures are often likened to a ‘black box’ in reproductive biology, partly due to the tissue's cellular complexity and the technical challenges associated with conducting dynamic, multi‐element analyses within the intact structure of the testes.

The *Drosophila* melanogaster testis serves as a well‐established model organism for investigating germ cell differentiation and the associated cellular architecture [3, 4]. Within this system, the shift from mitotic cell division to meiosis is intricately governed by factors such as Bam [5]. Disruptions in this regulatory network can result in spermatogenic arrest and pathological conditions [6, 7]. Moreover, the individualization complex (IC) is involved in the formation and clearance of RBs during spermiogenesis, moving distally along the spermatid bundle in Drosophila [8]. In our investigation of metabolic regulation, we specifically examined cytochrome P450 enzymes, recognized for their influence on lipid balance and mitochondrial function [9, 10]. We demonstrate that perturbation of *Cyp4ae1* in *Drosophila* induces a striking spermatogonial differentiation arrest. Intriguingly, the resulting arrested germ cell aggregates morphologically resemble the cytoplasmic RBs formed during normal spermiogenesis, suggesting a potential mechanistic link between differentiation blockage and RB structure formation. Unraveling the compositional and metabolic identity of these pathological aggregates, and comparing them to their normal RB counterparts, is therefore crucial for understanding the metabolic basis of differentiation failure and associated male infertility.

Nonetheless, this endeavor encounters a significant technical obstacle. Traditional omics methodologies (including genomics, transcriptomics, and proteomics) lack the spatial resolution and multiplexing capacity to simultaneously map the diverse array of metabolites, lipids, and proteins within these distinct 3D structures inside intact tissue [11, 12]. These methods are frequently invasive, reliant on labeling, or incapable of offering a comprehensive chemical overview. This limitation has left the in situ biochemical landscape of both typical RBs and disease‐associated aggregates largely opaque, hindering progress from morphological observation to mechanistic insight.

To break through this barrier, we developed the Super‐multiplexed Label‐free Raman Imaging (SLRI) as a transformative analytical platform. The term ‘super‐multiplexed’ was previously introduced in Raman‐based imaging to describe highly multiplexed detection enabled by engineered Raman tags or spectrally derived strategies, allowing for the simultaneous imaging of 20 or more distinct molecular channels [13]. SLRI leverages vibrational spectroscopy to generate high‐resolution, label‐free 2D/3D reference‐constrained spatial maps directly in unperturbed tissue. It enables the simultaneous, in situ detection of proteins, lipids, and other key metabolites without the need for staining or sectioning. Applying SLRI to the *Drosophila* testis, we have achieved the first direct visualization and compositional mapping of the two‐stage RB formation process during normal spermiogenesis. Furthermore, we deploy this platform to decipher the aberrant metabolic remodeling—including dysregulated lipid and mitochondrial metabolism—within the *Cyp4ae1*‐induced, differentiation‐arrested aggregates. By bridging a critical technological gap, this work establishes SLRI as a versatile tool that provides unprecedented insights into the metabolic reprogramming underlying cytoplasmic RB formation and pathological spermatogonia differentiation arrest.

## 2 Results

### 2.1 SLRI Enables Three‐dimensional Molecular Imaging of *Drosophila* Testes

**FIGURE 1:**
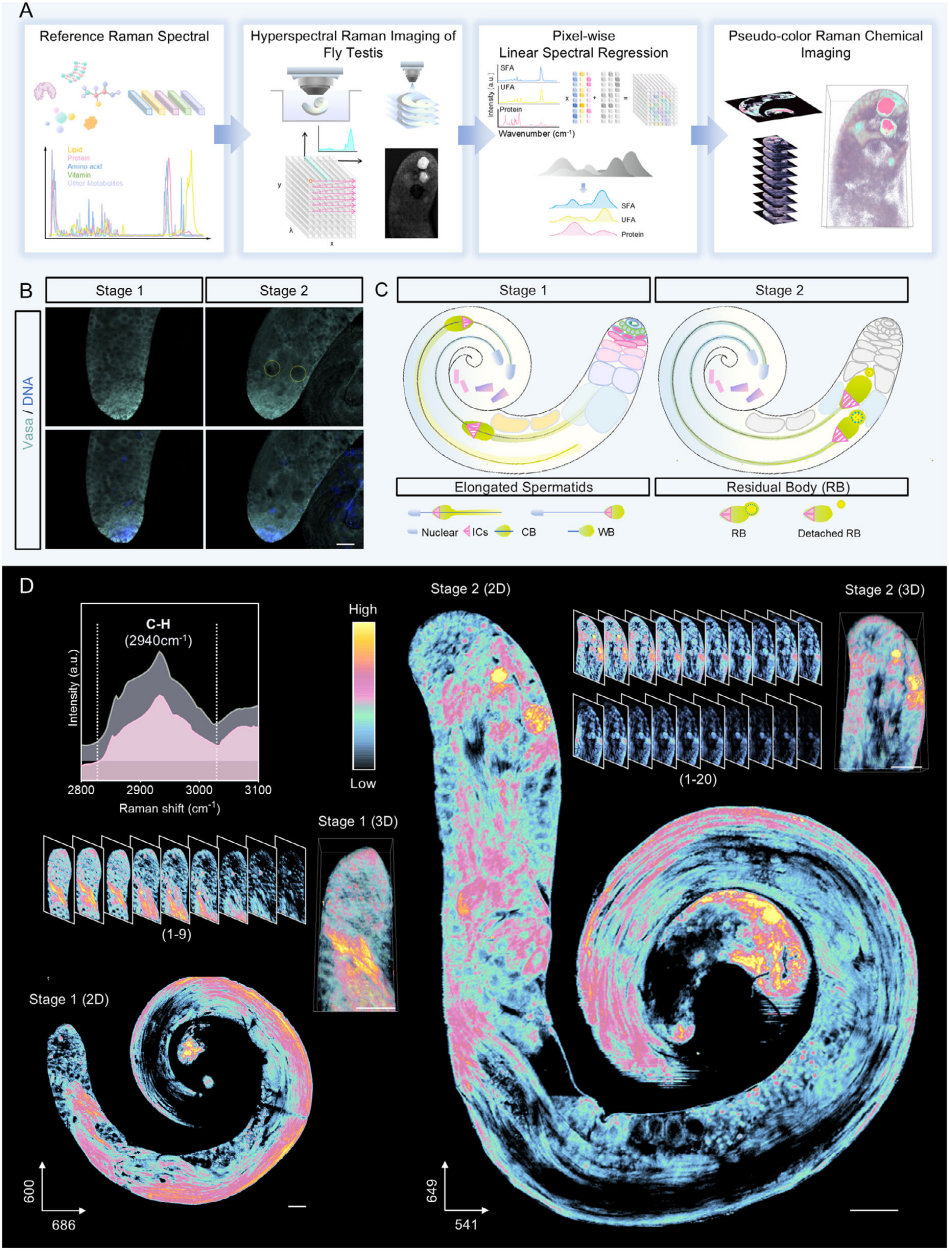
SLRI analysis in *Drosophila* testes. (A) Scheme of the testicular SLRI analysis approach. SLRI combines hyperspectral spontaneous Raman spectroscopy and spectral regression, employing NNLS to deconvolute complex signals into distinct biomolecular signatures. (B) Immunostaining of Vasa for germ cells in Stage 1 and Stage 2 testes. DNA was stained with Hoechst33342. (C) Schematic diagram of testes for cytoplasmic residual body (RB) in Stage 1 and Stage 2 testes. (D) 2D/3D Raman imaging of C─H bonds for Stage 1 and Stage 2 testes. The color shown at the top of the colorbar represents relatively higher signal intensity. Scale bar: 50 µm.

To dissect the biochemical basis of spermatogenesis and cytoplasmic RB dynamics in situ, we developed label‐free spectral Raman imaging, a method (SLRI) that integrates hyperspectral spontaneous Raman spectroscopy with spectral regression. This approach converts a single confocal Raman hyperspectral data cube (X‐Y‐Z‐λ) into spatially resolved molecular maps via reference‐based regression (Figure 1A). We first acquired full Raman spectra (300–3 800 cm^−^^1^) and constructed a comprehensive spectral library covering major biomolecular classes, including lipids, metabolites, and proteins.

During long‐term observation, we identified two distinct testicular stages: Stage 1, where Vasa‐positive germ cells fully occupy the apical testis, and Stage 2, characterized by partial loss of Vasa signal and the emergence of cytoplasmic RBs (Figure 1B,C). Applying SLRI to whole‐mount *Drosophila* testes, we generated global 2D molecular images and performed 3D volumetric reconstructions of the apical region, enabling unbiased visualization of testicular architecture and RB distribution (Figure 1D). SLRI‐based imaging of C─H bond distribution revealed that Vasa‐depleted areas in Stage 2 are populated by RBs (Figure 1D). Notably, in Stage 1, spermatid tails formed partial RBs, whereas in Stage 2, we observed both attached and detached RBs (Figure 1D). These findings establish SLRI as a powerful tool for 3D molecular phenotyping of intact testes and provide the first spatial evidence linking RB accumulation to germ cell loss.

### 2.2 SLRI Reveals the Compositional Landscape and Metabolic Compartmentalization of Cytoplasmic RBs

**FIGURE 2:**
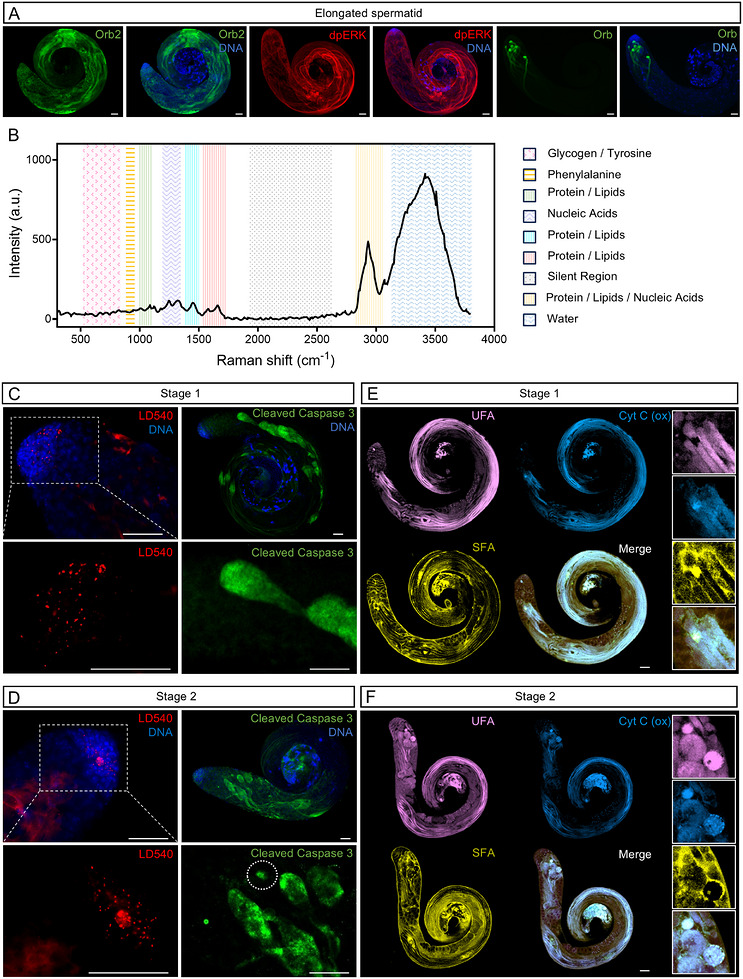
Identification of cytoplasmic RBs. (A) Immunostaining of Orb2, dpERK and Orb in testes, displaying different stages of elongated spermatids. DNA was stained with Hoechst33342. (B) The continuous spectral window between 300 and 3800 cm^−^^1^. (C, D) Lipid droplet (LD540) staining and immunostaining of cleaved Caspase 3 in Stage 1 (C) and Stage 2 (D) testes. DNA was stained with Hoechst33342. (E, F) Raman imaging reveals distributions of UFA, SFA and Cyt C ox for the entire *Drosophila* testes from Stage 1 (E) and Stage 2 (F). Scale bar: 50 µm.

To characterize the molecular composition of cytoplasmic RBs, we began by examining established germ cell markers via immunofluorescence. The results showed distinct localization patterns: Orb2 was broadly distributed throughout early‐stage germ cells and the spermatid tail, dpERK was primarily expressed in the spermatid tail, while Orb protein exhibited a strong and specific expression pattern within the tail end of spermatids and RBs (Figure 2A). Notably, dpERK partially colocalized with Orb2, but did not colocalize with Orb signal (Figure S1A,B), suggesting the existence of functionally distinct sub‐compartments within elongated spermatids.

We next applied SLRI to probe the biochemical basis of RBs in an unbiased, label‐free manner. This approach provides a continuous hyperspectral window (300–3 800 cm^−^^1^), enabling comprehensive assessment of testicular constituents, including diverse lipid subtypes, proteins, and metabolites that are often underrepresented in conventional analyses (Figure 2B). Using cleaved Caspase 3, we identified both cystic bulges (CBs) and waste bags (WBs) during RB morphogenesis in elongated spermatids (Figure 2C,D). Notably, detached cytoplasmic RBs were observed at the termini of elongated spermatids in Stage 2 testes (Figure 2D). Furthermore, lipophilic dye LD540 staining revealed a progressive accumulation and eventual clustering of lipid droplets from Stage 1 to Stage 2 (Figure 2C,D).

To systematically map the biomolecular makeup of RBs, we performed SLRI‐based chemical imaging across the testis. Strikingly, unsaturated fatty acids (UFA) and oxidized cytochrome c [Cyt C (ox)] showed pronounced localization to elongated spermatids and RBs, whereas saturated fatty acids (SFA) were not observed in these compartments (Figure 2E,F). These specific accumulations highlight RBs as metabolically specialized compartments enriched (i.e., showing relatively higher distribution) in unsaturated lipids and redox‐active molecules, which may support the biosynthetic demands of rapid spermatogonial divisions. We next performed quantitative analysis on representative signals using the original unmixing intensities of UFA, SFA, and Cyt C (ox). The results showed an overall increasing trend of these signals in Stage 2 testes (Figure S1C–E). This quantitative analysis reflects global signal intensity changes across testis samples and may not fully capture local variations within specific structures such as RB regions. Therefore, our study is mainly focused on the in situ spatial distribution and remodeling of molecular components within specific regions of the testis, and the biological conclusions are primarily derived from these spatially resolved patterns rather than global intensity changes.

### 2.3 A Spermatogonial Differentiation Arrest Model Recapitulates Metabolic and Subcellular Features of RBs

**FIGURE 3:**
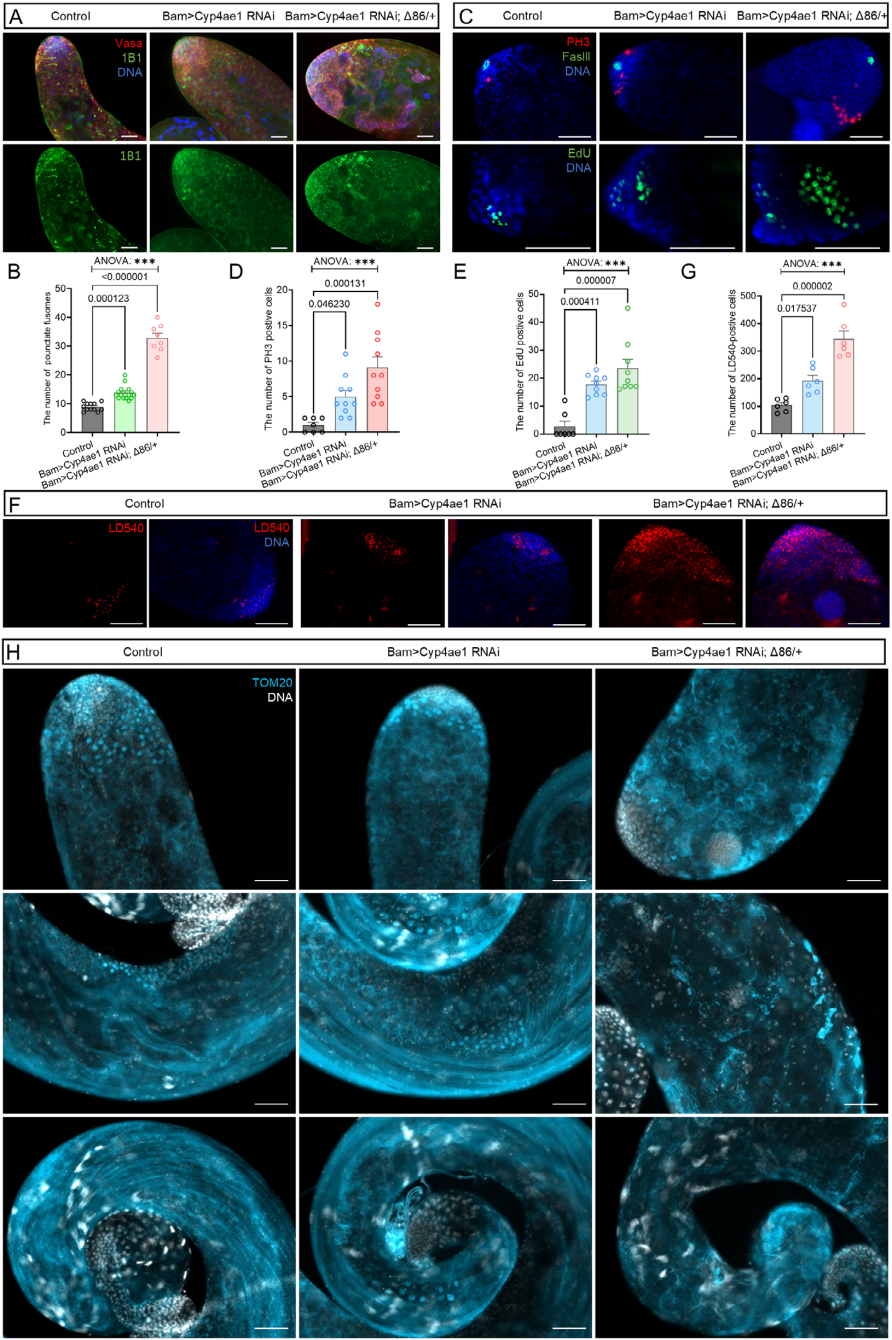
Cyp4ae1 is required for spermatogonia differentiation in testes. (A) Immunostaining of Vasa and 1B1 in control, *Bam>Cyp4ae1 RNAi* and *Bam>Cyp4ae1 RNAi; Δ86/+* groups. (B) The number of 1B1‐positive punctate fusomes. (C) Immunostaining of PH3 and EdU in control, *Bam>Cyp4ae1 RNAi* and *Bam>Cyp4ae1 RNAi; Δ86/+* groups. FasIII labels hub cells. (D) The number of PH3‐positive cells. (E) The number of EdU‐positive cells. (F) Staining of LD540 in control, *Bam>Cyp4ae1 RNAi* and *Bam>Cyp4ae1 RNAi; Δ86/+* groups. (G) The number of LD540‐positive cells. (H) Immunostaining of TOM20 in control, *Bam>Cyp4ae1 RNAi* and *Bam>Cyp4ae1 RNAi; Δ86/+* groups. DNA was stained with Hoechst33342. *P* < 0.05 was considered statistically significant. Scale bar: 50 µm.

To establish a tractable model for probing the substance basis of differentiation arrest—and to test whether arrested spermatogonia share metabolic properties with RBs—we generated the spermatogonia‐specific knockdown assay of *Cyp4ae1*, which encodes a key CYP450 enzyme subunit involved in monooxygenation reactions critical for diverse substrate metabolism [14]. To knock down *Cyp4ae1* in testicular spermatogonia, we employed the *Bam‐Gal4/UAS* system with two distinct UAS‐RNAi transgenic lines targeting different sequences. The knockdown efficiency was subsequently validated by qRT‐PCR (Figure S2A,B). Immunostaining for Vasa (germ cells) and 1B1 (fusomes) revealed a significant increase in punctate 1B1‐positive fusomes in *Bam>Cyp4ae1 RNAi* and *Bam>Cyp4ae1 RNAi‐II* testes compared to controls, indicating a blockade in spermatogonial differentiation (Figure 3A,B and Figure S2C,D). This phenotype was markedly enhanced in a heterozygous *bam* mutant background (*Δ86/+*), which produced large aggregates of undifferentiated germ cells (Figure 3A,B and Figure S2C,D). Consistent with a proliferative arrest, phospho‐Histone H3 (PH3) and EdU labeling showed increased numbers of proliferating cells in *Cyp4ae1 RNAi* testes, with further elevation in the *Δ86/+* background (Figure 3C–E and Figure S2E, F). Given the established role of CYP450 enzymes in lipid homeostasis and mitochondrial function, we examined metabolic and organellar alterations in the arrest model. LD540 staining demonstrated substantial accumulation of lipid droplets in *Bam>Cyp4ae1 RNAi* and *Bam>Cyp4ae1 RNAi‐II* testes, which was further intensified by the *Δ86/+* background (Figure 3F–G and Figure S2G–H). Mitochondrial integrity, assessed by TOM20 staining, was progressively disrupted during spermatogenesis in *Bam>Cyp4ae1 RNAi* testes, with severe defects initiating at the spermatogonial stage in the *Δ86/+* background (Figure 3H). Collectively, these data validate a functional differentiation‐arrest model that recapitulates key features observed in RB‐rich regions—including lipid droplet accumulation and mitochondrial perturbation—thereby providing a genetically defined system for subsequent Raman‐based biochemical imaging.

### 2.4 Convergent Metabolic Signatures in RBs and Arrested Spermatogonia

**FIGURE 4:**
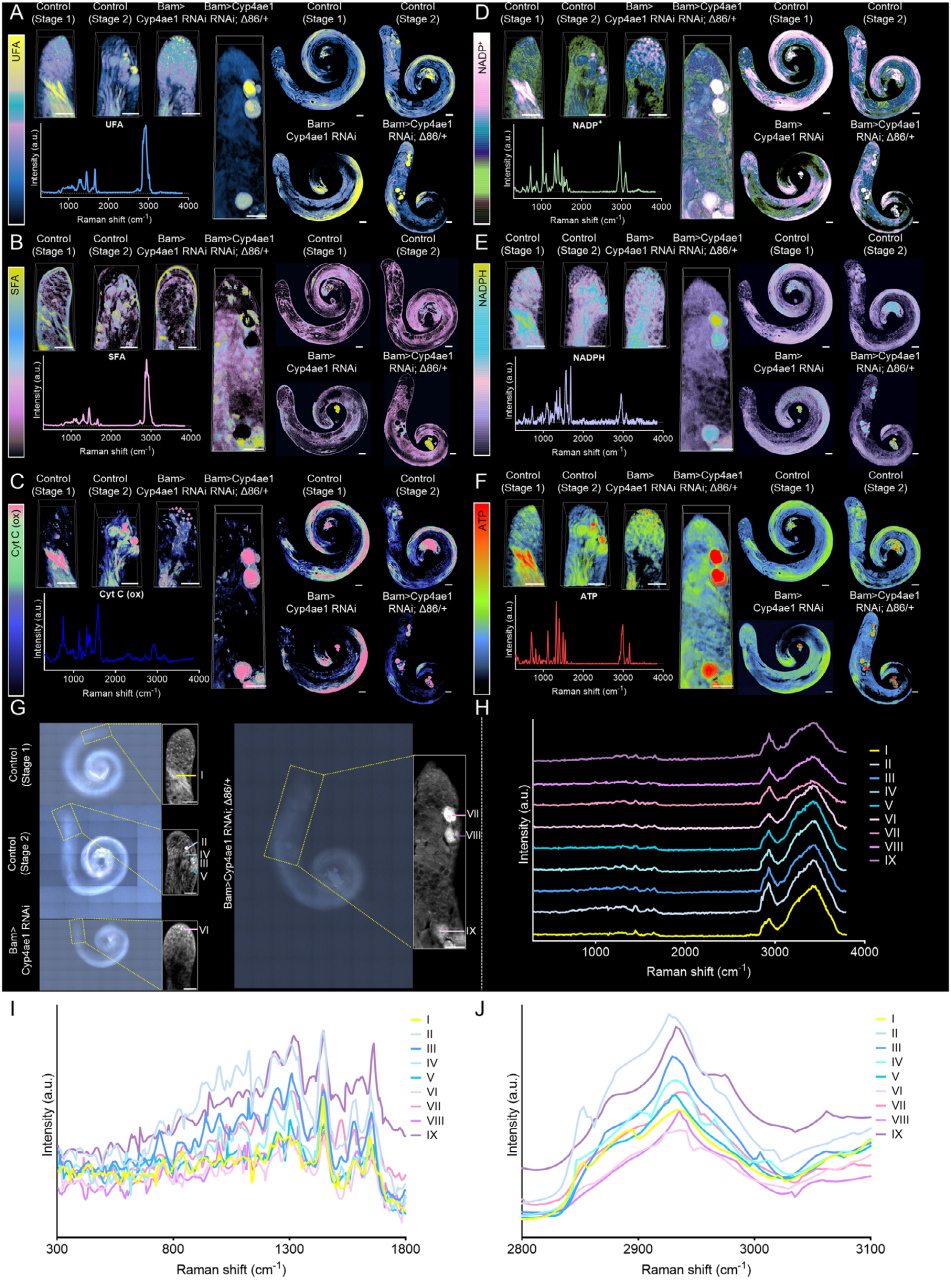
Substance comparisons in cytoplasmic RBs and arrested spermatogonia. (A–F) Raman imaging reveals 2D/3D distributions of UFA (A), SFA (B), Cyt C (ox) (C), NADP+ (D), NADPH (E), and ATP (F) in testes. (G) Comparison of substance similarity in control Stage 1 RBs (I); control Stage 2 detached RBs (II, III); control Stage 2 attached RBs (IV, V); arrested spermatogonia from *Bam>Cyp4ae1 RNAi* testes (VI); and early‐, mid‐, and late‐stage arrested clusters from *Bam>Cyp4ae1 RNAi; Δ86/+* testes (VII–IX). (H) Spectrum features (300–3 800 cm^−^^1^) from nine defined anatomical regions (I∼IX). (I, J) Spectrum characteristics of specific segments, e.g. 300–1 800 cm^−^^1^ (I) and 2 800–3 100 cm^−^^1^ (J) from nine defined anatomical regions (I∼IX). The color shown at the top of the colorbar represents relatively higher signal intensity. Scale bar: 50 µm.

Given the structural resemblance between arrested spermatogonial clusters and cytoplasmic RBs, we employed 2D and 3D Raman spectral imaging to perform a comparative, in situ analysis of their metabolic compositions across the apical testis of control (Stage 1/2), *Bam>Cyp4ae1* RNAi, and *Bam>Cyp4ae1 RNAi; Δ86/+* groups. Strikingly, arrested spermatogonia in both *Bam>Cyp4ae1* RNAi testes and the more severe *Bam>Cyp4ae1 RNAi; Δ86/+* model recapitulated the key metabolic signature of RBs: a pronounced heterogeneous spatial intensity pattern of UFA and Cyt C (ox), coupled with a consistent absence of SFA (Figure 4A–C and Video S1). Within the spatially organized arrested clusters—where distance from the apex serves as a proxy for differentiation age—we observed a dynamic biochemical progression. This was characterized by persistently high Cyt C (ox) levels, sustained absence of SFA, and a relatively spatially decreased UFA signal from early to late‐stage clusters (Figure 4A–C). Furthermore, NADP^+^, NADPH, and ATP were relatively spatially co‐enriched in both cytoplasmic RBs and regions of arrested spermatogonia (Figure 4D–F and Video S1). Notably, NADPH intensity within arrested clusters decreased over time (Figure 4E). Consistent with mitochondrial dysfunction, *Cyp4ae1*‐deficient testes exhibited an increased NADP^+^/NADPH ratio and elevated ATP levels compared to controls (Figure S3). However, ATP and NADP^+^/NADPH measurements from the bulk testicular lysates reflect overall metabolic status and cannot be directly interpreted as RB‐specific metabolic composition. In contrast, SLRI enables in situ analysis of metabolic distributions without the need for physical isolation of subcellular structures. Therefore, in this study, SLRI is employed to provide spatially resolved evidence of metabolic remodeling in RB regions, while bulk biochemical assays serve as complementary validation for overall metabolic changes.

To obtain comprehensive spectral fingerprints, we collected Raman spectra (300–3 800 cm^−^^1^) from nine defined anatomical regions (Figure 4G): Stage 1 RBs (I); Stage 2 detached RBs (II, III); Stage 2 attached RBs (IV, V); arrested spermatogonia from *Bam>Cyp4ae1 RNAi* testes (VI); and early‐, mid‐, and late‐stage arrested clusters from *Bam>Cyp4ae1 RNAi; Δ86/+* testes (VII–IX). Regions I–IX were primarily selected to investigate the substance similarity and heterogeneity between RBs and arrested spermatogonia. Broad‐spectrum and narrow‐band analyses revealed both shared spectral features and region‐specific signatures (Figure 4H–J and Figure S4), highlighting the metabolic similarity between RBs and differentiation‐arrested germ cells. Finally, application of the SLRI algorithm resolved finer metabolic subtypes in situ, uncovering a heterogeneous biochemical landscape underlying the arrested state.

### 2.5 SLRI Maps Oxidative Stress, TCA Cycle Flux, Glycolytic Remodeling and Lipid Rewiring in RBs and Arrested Spermatogonia

**FIGURE 5:**
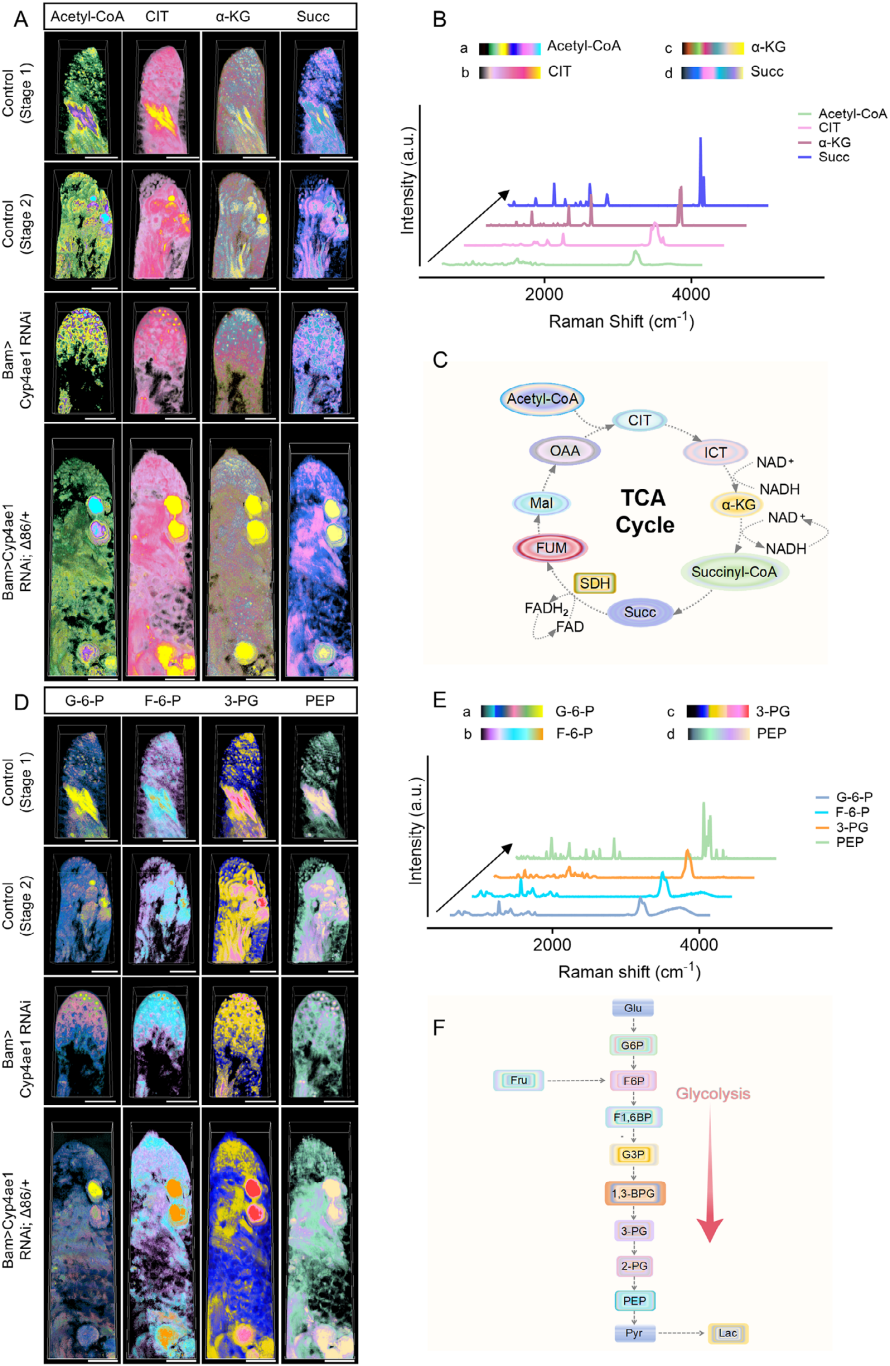
Substances for mitochondria and glycolysis in cytodynamic RBs and arrested spermatogonia. (A) Raman imaging reveals 3D distributions of Acetyl‐CoA, CIT, α‐KG, and Succ in Stage 1 (control), Stage 2 (control), *Bam>Cyp4ae1 RNAi* and *Bam>Cyp4ae1 RNAi; Δ86/+* testes. (B) Raman spectra of Acetyl‐CoA, CIT, α‐KG, and Succ standards. (C) Schematic diagram of TCA metabolism. (D) Raman imaging reveals 3D distributions of G‐6‐P, F‐6‐P, 3‐PG and PEP in Stage 1 (control), Stage 2 (control), *Bam>Cyp4ae1 RNAi* and *Bam>Cyp4ae1 RNAi; Δ86/+* testes. (E) Raman spectra of G‐6‐P, F‐6‐P, 3‐PG and PEP standards. (F) Schematic diagram of glycolysis metabolism. The color shown at the right end of the colorbar represents relatively higher signal intensity. Scale bar: 50 µm.

To elucidate how cellular structure and metabolic state interact during differentiation arrest, we performed systematic Raman imaging to profile a broad spectrum of metabolites within cytoplasmic RBs and arrested spermatogonia. Both compartments accumulated key oxidative stress markers, including oxidized and reduced glutathione (GSSG and GSH), superoxide dismutase (SOD), 8‐Hydroxy‐2'‐deoxyguanosine (8‐OHdG), 8‐iso Prostaglandin F2alpha (8‐iso PGF2α), and S‐lactoylglutathione (S‐LG), indicative of active redox remodeling (Figure S5 and Video S2). Notably, GSSG and GSH were simultaneously relative spatially enriched, suggesting a complex, regulated oxidative stress response (Figure S4A,B). Concomitantly, TCA cycle intermediates—acetyl‐CoA (Acetyl‐CoA), citrate (CIT), α‐ketoglutarate (α‐KG) and succinate (Succ)—were strongly elevated in RBs and arrested spermatogonia (Figure 5A–C and Video S3). Acetyl‐CoA and Succ displayed marked spatially decreased distributions from early to late arrested clusters, reflecting a dynamic metabolic progression within the arrested state (Figure 5A).

Glycolytic metabolites also exhibited a prominent, spatially enriched distribution. Glucose‐6‐phosphate (G‐6‐P), fructose‐6‐phosphate (F‐6‐P), 3‐phosphoglycerate (3‐PG), phosphoenolpyruvate (PEP), fructose (Fru), and pyruvate (Pyr) all accumulated in RBs and arrested spermatogonia (Figure 5D–F; Figure S6 and Video S3). While pyruvate remained consistently high, G‐6‐P, F‐6‐P, 3‐PG, PEP, and Fru declined specifically in late‐stage arrested clusters, pointing to a temporal shift in glycolytic flux during prolonged arrest.

**FIGURE 6:**
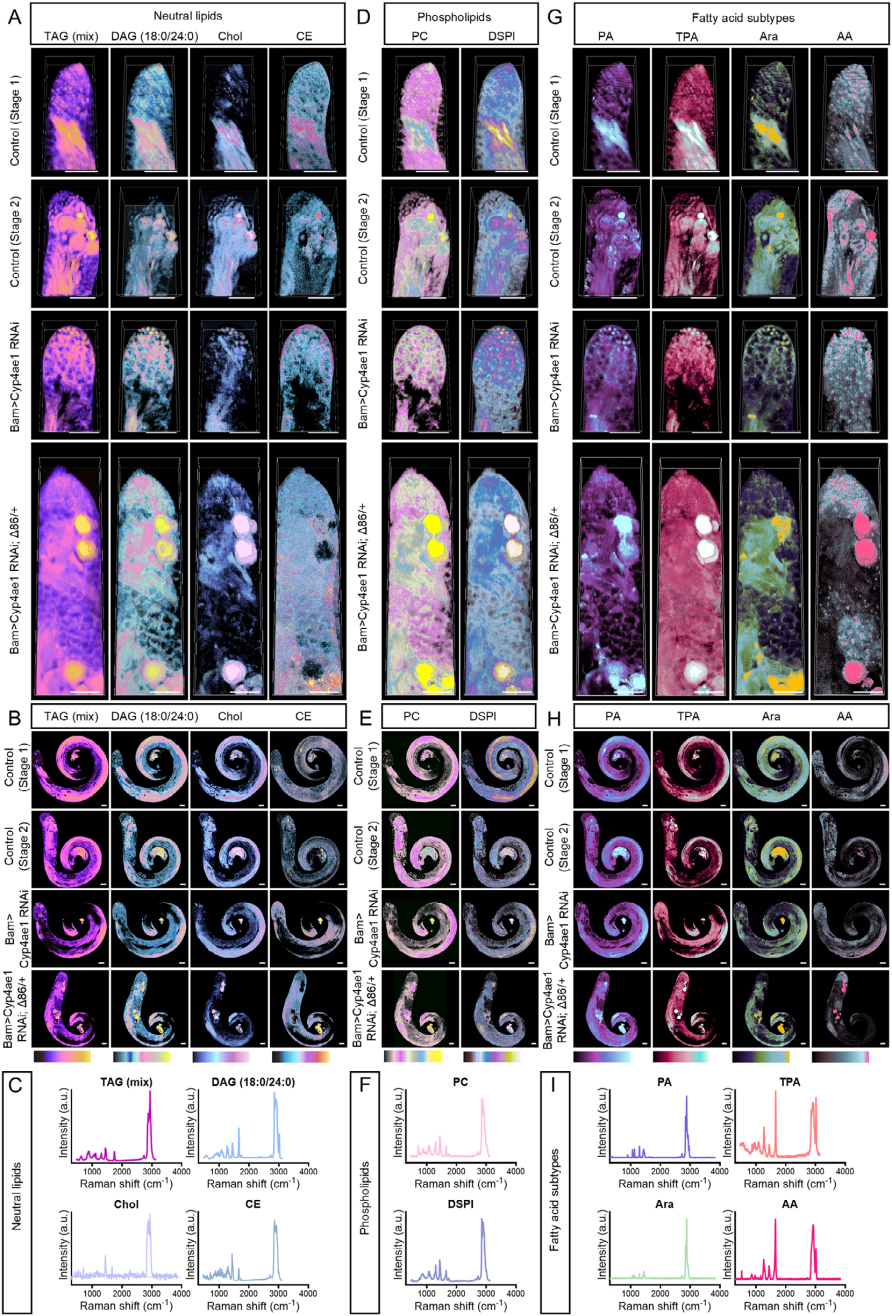
Substances for lipid metabolism in cytodynamic RBs and arrested spermatogonia. (A) Raman imaging reveals 3D distributions of neutral lipids, including TAG mix, DAG 18:0/24:0, Chol and CE in Stage 1 (control), Stage 2 (control), *Bam>Cyp4ae1 RNAi* and *Bam>Cyp4ae1 RNAi; Δ86/+* testes. (B) Raman imaging reveals 2D distributions of TAG mix, DAG 18:0/24:0, Chol and CE in corresponding testes. (C) Raman spectra of TAG mix, DAG 18:0/24:0, Chol and CE standards. (D) Raman imaging reveals 3D distributions of phospholipids (PC and DSPI) in Stage 1 (control), Stage 2 (control), *Bam>Cyp4ae1 RNAi* and *Bam>Cyp4ae1 RNAi; Δ86/+* testes. (E) Raman imaging reveals 2D distributions of PC and DSPI in corresponding testes. (F) Raman spectra of PC and DSPI standards. (G) Raman imaging reveals 3D distributions of representative fatty acid subtypes (PA, TPA, Ara and AA) in Stage 1 (control), Stage 2 (control), *Bam>Cyp4ae1 RNAi* and *Bam>Cyp4ae1 RNAi; Δ86/+* testes. (H) Raman imaging reveals 2D distributions of PA, TPA, Ara and AA in corresponding testes. (I) Raman spectra of PA, TPA, Ara and AA standards. The color shown at the right end of the colorbar represents relatively higher signal intensity. Scale bar: 50 µm.

Striking lipid rewiring was observed. Neutral lipids—including mixed triacylglycerols (TAG Mix), diacylglycerol (DAG) 18:0/24:0, and cholesterol (Chol)—markedly increased in both compartments (Figure 6A–C). This pattern suggests a shift toward lipid storage, yet with impaired conversion of Chol to its storage form, cholesterol ester (CE) in arrested clusters, where the CE signature was not virtually detectable (Figure 6A–C and Video S4). Concurrently, key phospholipids—phosphatidylcholine (PC) and distearoyl phosphatidylinositol (DSPI)—were significantly elevated (Figure 6D–F and Video S4), indicating active membrane remodeling alongside storage. Fatty acids profiling further revealed compartment‐specific changes: palmitic acid (PA), tetracosapentaenoic acid (TPA), arachidic acid (Ara), arachidonic acid (AA), and docosahexaenoic acid (DHA) all showed altered abundance, underscoring the diversity of lipid metabolic adjustments in RBs and arrested spermatogonia (Figure 6G–I and Video S4).

We further attempted to support SLRI‐observed metabolic changes using non‐targeted metabolomics in control, *Bam>Cyp4ae1 RNAi*, and *Bam>Cyp4ae1 RNAi; Δ86/+* testes. Overall, we identified 52 up‐regulated and 13 down‐regulated differential metabolites between control and *Bam>Cyp4ae1 RNAi* testes, as well as 35 up‐regulated and 24 down‐regulated differential metabolites between control and *Bam>Cyp4ae1 RNAi; Δ86/+* testes (Figure S7A). We also note that non‐targeted metabolomics provides limited coverage of metabolite identities and often detects derivative or fragmented signals, which are not always directly comparable to Raman‐based molecular assignments. The heatmap further illustrated the alteration patterns of lipids and lipid‐like molecules in both comparisons (Figure S7B, C), which revealed a certain degree of similarity to the lipid metabolic trends observed in our SLRI analysis.

Spatial analysis revealed distinct lipid distribution patterns within RBs. TAG Mix, DAG 18:0/24:0, Chol, CE, PC, TPA, PA, and Ara formed a diffuse central region surrounded by a ring‐like accumulation of punctate particles at the periphery (Figure S8). Raman spectra acquired from peripheral puncta (loci a‐c) and the central diffuse region (loci d‐f) demonstrated a certain degree of similarity, suggesting shared biochemical spectral features despite distinct spatial organization (Figure S9).

Together, these data exhibit overlapping Raman‐inferred biochemical profiles in RBs and arrested spermatogonia, encompassing oxidative stress management, sustained TCA and glycolytic activity, and a profound rewiring of lipid storage and membrane composition.

### 2.6 SLRI Reveals Accumulation and Compartmentalization of Amino Acids and Vitamins in RBs and Arrested Spermatogonia

**FIGURE 7:**
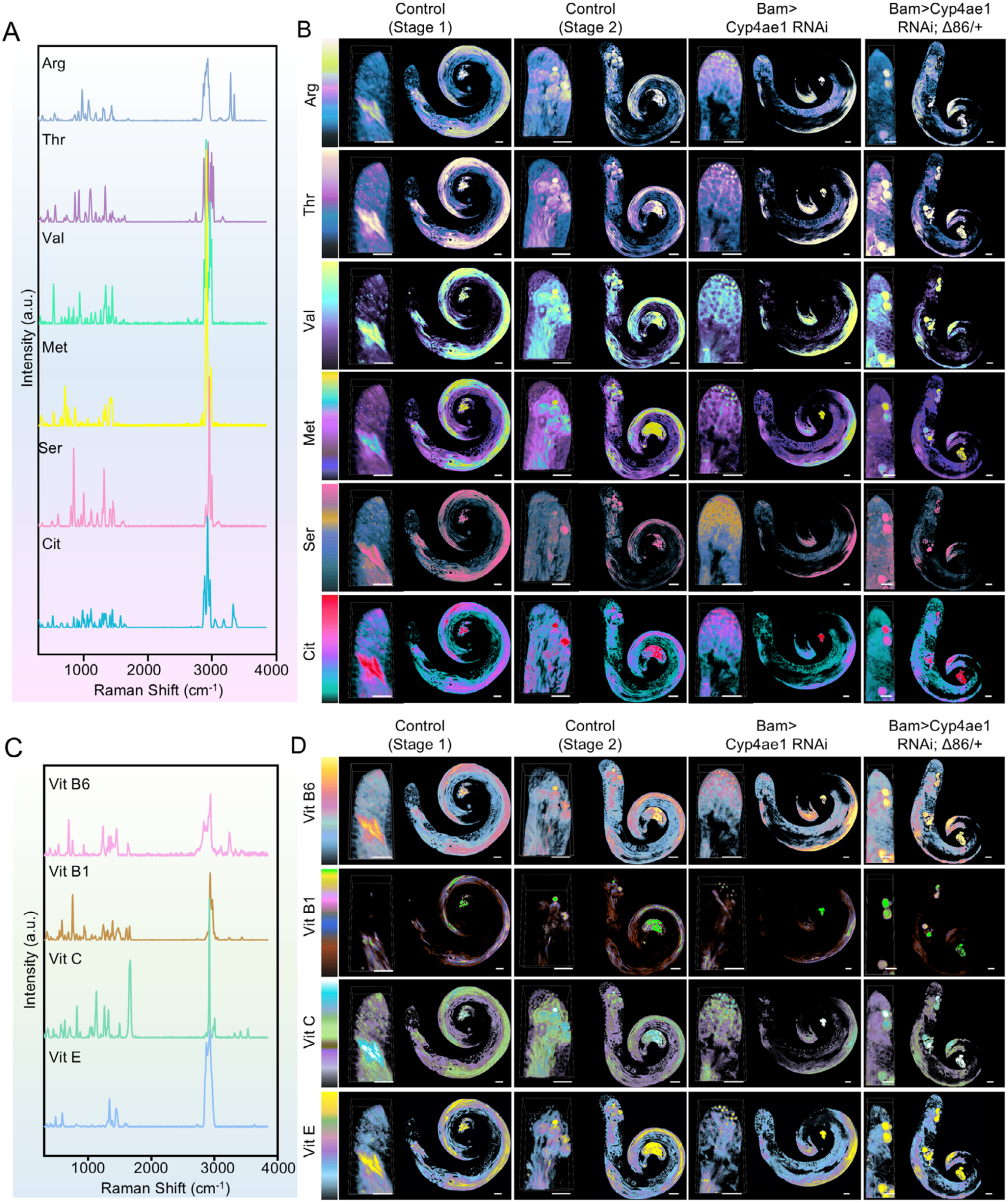
Substances for amino acids and vitamins in cytodynamic RBs and arrested spermatogonia. (A) Raman spectra of Arg, Thr, Val, Met, Ser and Cit standards. (B) Raman imaging reveals 2D/3D distributions of Arg, Thr, Val, Met, Ser and Cit in Stage 1 (control), Stage 2 (control), *Bam>Cyp4ae1 RNAi* and *Bam>Cyp4ae1 RNAi; Δ86/+* testes. (C) Raman spectra of Vit B6, Vit B1, Vit C and Vit E standards. (D) Raman imaging reveals 2D/3D distributions of Vit B6, Vit B1, Vit C and Vit E in Stage 1 (control), Stage 2 (control), *Bam>Cyp4ae1 RNAi* and *Bam>Cyp4ae1 RNAi; Δ86/+* testes. The color shown at the top of the colorbar represents relatively higher signal intensity. Scale bar: 50 µm.

Amino acids and vitamins are well‐established essential nutrients vital for spermatogenesis [10, 15]. Yet, their distribution within specific structures like cytoplasmic RBs, particularly in pathological states such as arrested spermatogonia, remains largely unexplored. To extend metabolic profiling to under‐explored molecule classes, we applied SLRI to map the distribution of amino acids and vitamins within *Drosophila* testes, focusing on cytoplasmic RBs and arrested spermatogonia. Strikingly, six amino acids—L‐arginine (Arg), L‐threonine (Thr), L‐valine (Val), L‐methionine (Met), L‐serine (Ser), and L‐citrulline (Cit)— were relatively spatially enriched in both RBs and arrested spermatogonia compared to surrounding cells (Figure 7A,B and Video S5). Moreover, based on our non‐targeted metabolomics data from control, Bam>Cyp4ae1 RNAi, and Bam>Cyp4ae1 RNAi; Δ86/+ testes, we also identified several differential metabolites associated with amino acid metabolism. These metabolites predominantly showed an upward trend, and overall exhibited a certain degree of similarity with the SLRI findings (Figure S10).

A parallel accumulation was observed for four vitamins (Figure 7C,D and Video S6): vitamin B6 (Vit B6), vitamin B1 (Vit B1), vitamin C (Vit C) and vitamin E (Vit E). Within arrested spermatogonial clusters, Arg, Met, Cit, Vit B6, Vit B1 and Vit C displayed a marked, spatially decreased signal from early to late stages (Figure S11), suggesting their active consumption or turnover during sustained arrest.

Remarkably, several of these metabolites—including Arg, Thr, Met, Cit, Vit B6, Vit B1, Vit C and Vit E—exhibited a bipartite spatial organization within RBs: a diffuse signal in the central region surrounded by a ring‐like accumulation of scattered punctate particles at the periphery (Figure S12). This pattern closely mirrors the lipid distribution described earlier (Figure S8), indicating a shared principle of spatial compartmentalization for diverse biomolecules. Together, these data establish that RBs and arrested spermatogonia serve as local reservoirs for amino acids and vitamins, and reveal a conserved, spatially organized architecture for their storage. These findings provide the first in situ landscape of amino acid and vitamin compartmentalization in the testis, offering critical insights into their potential roles for RB formation and spermatogonia differentiation arrest.

## 3 Discussion

For over a century, the precise biochemical composition of cytoplasmic RBs during spermiogenesis has remained unresolved [16, 17], representing a fundamental gap in reproductive biology. Here, we leveraged our newly developed SLRI platform to provide the first high‐resolution, in situ Raman‐derived atlas for RB formation and spermatogonia differentiation arrest.

### 3.1 SLRI Reveals RB Composition and Dynamics in 3D

Our application of SLRI enabled unprecedented volumetric visualization of testicular tissue, revealing that RBs are not inert waste products but dynamic, metabolically active compartments enriched in lipids, proteins, and specific metabolites. The observed decrease in RB size concurrent with marked substance accumulation suggests a process of biochemical concentration and maturation, supporting the long‐standing hypothesis that RBs serve as a recycled substrate reservoir for spermatogenesis [18, 19].

It is worth mentioning that isolation of RBs for independent biochemical validation, such as mass spectrometry or targeted assays, remains technically challenging in the current system. In *Drosophila* testes, RBs are transient and highly sensitive structures formed during the late stage of spermiogenesis, and are difficult to preserve outside their native spatial context. In addition, approaches such as fluorescence‐activated cell sorting or density gradient centrifugation require enzymatic or mechanical dissociation of testicular tissue, which may disrupt RB integrity and lead to metabolite leakage, redistribution, or contamination from other cellular components. These factors limit the feasibility of obtaining RB‐enriched preparations with sufficient purity and yield for reliable downstream biochemical or mass spectrometric analyses. Robust quantification of structure‐specific molecular differences will require future development of reliable segmentation and micro‐region‐based analysis approaches.

### 3.2 Metabolic Similarity between RBs and Arrested Spermatogonial Clusters

By integrating observations with a cytochrome P450‐mediated spermatogenic dysfunction model [9, 20, 21], we identified a striking metabolic resemblance between normal RBs and clusters of arrested spermatogonia. Both structures exhibited pronounced signatures of altered lipid metabolism and mitochondrial activity. Evidence has established that oxidative stress impairs lipid metabolic homeostasis by facilitating lipid droplet accumulation [22, 23]. Notably, spatial mapping within arrested clusters revealed temporal heterogeneity in these metabolic pathways, suggesting that differentiation failure is associated with dysregulated, rather than simply arrested, metabolic remodeling. However, we only observed that RBs and *Cyp4ae1* RNAi‐induced arrested spermatogonial clusters exhibit overlapping Raman‐inferred biochemical profiles. This similarity reflects comparable biochemical compositions at the level of Raman‐detectable signals, while functional evidence for metabolite transfer or recycling is currently lacking. Future studies will be needed to further investigate and establish any potential causal relationship between RBs and arrested spermatogonial clusters.

### 3.3 Amino Acid and Vitamin Metabolism: An Underexplored Dimension of Germ Cell Biology

Beyond core energy metabolism, SLRI uniquely enabled the label‐free, in situ mapping of amino acids and vitamins—classes of molecules notoriously difficult to preserve and visualize with conventional spatial methods. This poses a challenge for the analysis of amino acid and vitamin‐related metabolic pathways, as water‐soluble and preparation‐sensitive metabolites are easily disrupted or depleted [24, 25]. Recent studies have employed Raman spectroscopy to assess the effects of exogenous amino acids at the cellular level [26]. Similarly, research on the Raman spectroscopy of vitamins remains limited, with only a few studies reporting on vitamin precursors like beta‐carotene [27], while others have explored the effects of vitamin supplementation [28]. Despite these advances, there is still a paucity of research on the in situ distribution of amino acid and vitamin subtypes in tissues, prompting us to further investigate this area. By using SLRI, we found that specific amino acids (e.g., Arg, Thr, Val, Met, Ser, Cit) and vitamins (Vit B6, Vit B, Vit C, Vit E) are selectively enriched within RBs and arrested spermatogonia. The rapid degradation of Arg, Met, Cit, Vit B6, Vit B1, and Vit C upon differentiation arrest points to their potential role as critical cofactors or signaling molecules in normal spermiogenesis. The observed spatiotemporal heterogeneity in their distribution opens a new avenue for investigating how localized nutrient availability and vitamin‐dependent pathways coordinate germ cell development.

Previous studies have shown that vitamins can participate in spermatogenesis not only as nutritional factors but also through metabolic and signaling pathways that regulate germ cell fate. For example, vitamin B12 has been reported to promote spermatogonial proliferation and reduce germ cell loss induced by busulfan treatment [29]. In addition, vitamin A and its active metabolite retinoic acid are well‐established regulators of spermatogonial differentiation and meiotic initiation [30, 31], acting through pathways involving key regulators such as Stra8 and CYP26B1, as well as retinoic acid receptor signaling components [32, 33, 34]. These studies provide a broader biological context supporting the importance of vitamin‐associated metabolic regulation in spermatogenesis. Nevertheless, our current study primarily focuses on the in situ spatial distribution and remodeling of vitamins in the testis. Based on the spatial metabolic mapping and existing literature, we propose these observations as hypotheses for future investigation. Further functional validation, including vitamin supplementation, pathway perturbation, or *Cyp4ae1* rescue experiments, will be required to determine whether these metabolic changes directly contribute to differentiation arrest.

### 3.4 Condensed States of Specific Components in the RB Organization

Based on the punctate, non‐membrane‐bound aggregation of diverse lipid species, amino acids, and vitamins around RBs, we hypothesize that the biophysical organization of RBs may be associated with liquid–liquid phase separation (LLPS) [35, 36]. This aligns with emerging paradigms of lipid droplet formation and mRNA granule assembly in germ cells [37, 38, 39]. Moreover, the interpretation of these punctate patterns is based on XY‐plane spatial distributions and is not dependent on Z‐axis sampling density. We propose that RBs may constitute a specialized, phase‐separated condensate that sequesters and spatially organizes metabolites, lipids, and proteins for targeted recycling or signaling. At this stage, this serves as a working hypothesis; future systematic studies are required to elucidate the underlying mechanisms.

### 3.5 Technological Advance and Biological Implications

Raman spectroscopy has become widely adopted as a non‐destructive monitoring technique, offering a rapid alternative to traditional methods such as chemical extraction and histological analysis [40]. This study establishes SLRI as a transformative platform for intact organs. By overcoming the limitations of label‐dependent, destructive, or low‐resolution methods, we present a workflow that preserves native tissue chemistry while yielding Raman‐inferred molecular signatures with 2D/3D resolution. The ability to simultaneously visualize glycolysis, lipid subspecies, mitochondrial metabolites, amino acids, and vitamins in situ addresses a longstanding technical barrier and sets a new standard for integrative metabolic phenotyping in intact tissues.

In this study, immunofluorescence staining provides reliable validation of cellular and subcellular structures, including RBs, lipid droplets, mitochondrial organization, and germ cell differentiation states. These markers establish a robust morphological and cellular framework for interpreting the spatial organization observed in the Raman imaging data. In contrast, the Raman spectral decomposition yields spatial distributions of multiple metabolite‐associated signals; however, these Raman‐inferred molecular features are not independently validated at the level of definitive molecular identity for each individual metabolite. Therefore, our Raman‐derived maps are now consistently described as spectrally inferred molecular distributions, emphasizing their role as model‐constrained biochemical representations within intact tissue.

## 4 Conclusion

In summary, our work moves the century‐old question of RB from morphological description to mechanistic insight. We demonstrate that the substance basis of RBs is phenocopied in spermatogonia differentiation arrest. The SLRI platform developed here not only clarifies the role of RBs but also provides a generalizable framework for investigating metabolic spatial biology across a wide range of developmental and disease models in reproductive and beyond.

## 5 Materials and Methods

### 5.1 Fly Strains and Crosses

All *Drosophila* melanogaster lines were reared on a standard corn syrup medium at 25°C under a relative humidity ranging from 40% to 60%. Male individuals from the *Bam‐Gal4; Δ86/+* strain were randomly paired with transgenic *UAS‐Cyp4ae1 RNAi* (#TH04967.N obtained from THFC or v103964 obtained from VDRC) virgin females and maintained at 25°C until eclosion. Male flies aged between two to three days with the specific genotype (*Bam>Cyp4ae1 RNAi* or *Bam>Cyp4ae1 RNAi; Δ86/+*) were chosen for subsequent analyses. The *w^1118^* strain served as the control in these experiments.

### 5.2 Immunostainings

Following dissection of *Drosophila* testes in 1× phosphate‐buffered saline (PBS), they were fixed in 4% paraformaldehyde (PFA) for 30 min, washed thrice with 0.3% PBS‐Triton X‐100 (PBST), and then subjected to a 30 min blocking step in 5% bovine serum albumin (BSA). Subsequently, primary antibodies were administered to the testes and allowed to incubate for 1 h at room temperature. Testes were then washed three times in 0.3% PBST and incubated in the dark with secondary antibodies for 1 h at room temperature. After three additional rounds of washing with 0.3% PBST, testes were stained with Hoechst33342 (Solarbio, 1.0 mg/mL) for 5 min before being mounted. The primary antibodies used included rabbit anti‐Vasa (1:2000, a gift from Prof. Chao Tong, Zhejiang University), mouse anti‐Orb (DSHB, 1:50), mouse anti‐Orb2 (DSHB, 1:50), mouse anti‐1B1 (DSHB, 1:50), mouse anti‐FasIII (DSHB, 1:50), rabbit anti‐dpERK (#4370, CST, 1:200), rabbit anti‐PH3 (#53348, CST, 1:600), rabbit anti‐cleaved Caspase‐3 (#9664, CST, 1:200), and rabbit anti‐TOM20 (#42406, CST, 1:200). Secondary antibodies labeled with Cy3 or A647 (Jackson ImmunoResearch Laboratories) were diluted at a ratio of 1:400.

### 5.3 Lipid Droplet Assays

To assess the quantity and distribution of lipid droplets, the LD540 dye (C2050S, Beyotime) was employed. Testicular tissues were fixed in 4% PFA for 30 min. Following three rinses in 1× PBS, testes were treated with a solution comprising LD540 (1:2000) and Hoechst33342 (1:1000) in the dark for 20 min. Subsequently, they were washed thrice with 1× PBS.

### 5.4 ATP Measurement

For the ATP level analysis, an ATP assay kit (S0026, Beyotime) was utilized. The procedure involved dissecting fly testes in 1× PBS, with 20 testes per sample added to 200 µL of lysis buffer. Following complete homogenization, the samples were centrifuged at 12 000×g for 5 min at 4°C, and the supernatant was collected. Subsequently, 100 µL of ATP detection working solution was added to the detection well and allowed to incubate for 5 min at room temperature. Next, 20 µL of each sample was swiftly added to the detection well and mixed. The ATP levels were measured using a microplate reader.

### 5.5 NADP+/NADPH Ratio Detection Assay

To determine the NADP+/NADPH ratio in testicular tissues, the Enhanced NADP+/NADPH Assay Kit (S0180S, Beyotime) was employed. Approximately 50 pairs of testis samples were combined in an EP tube, and 400 µL of enhanced NADP+/NADPH extraction solution was added for homogenization and lysis. Following lysis, the samples were centrifuged at 12 000×g, 4°C for 10 min, and the resulting supernatant was collected as the test samples. G6PDH working solution and NADPH standard were prepared as per the kit's guidelines. The sample, extraction solution, and standard were mixed and incubated at 37°C for 10 min. Subsequently, a colorimetric reagent was introduced for the reaction. After the reaction, the absorbance was measured at 450 nm, and the NADP+/NADPH ratio in the sample was calculated using a standard curve.

### 5.6 Non‐Targeted Metabolomics Analysis

Testes from each group were used for non‐targeted metabolomics analysis. Metabolites were identified by matching their accurate masses and MS/MS spectra against the mzCloud database, as well as the mzVault and MassList databases. Both positive ion mode (POS) and negative ion mode (NEG) were applied to maximize metabolite coverage and detection efficiency. Metabolites with a VIP score ≥ 1 and a *p*‐value < 0.05 were considered significantly different between groups.

### 5.7 Confocal Raman Microscopy

Testes were embedded in agarose gel and then submerged in a PBS solution. To ensure accurate biochemical identification, a detailed Raman spectral library was established. This library was created using purified reference standards obtained through commercial sources, as well as substances sourced from laboratory stocks (Table S1). DAG (18:0/24:0), also known as 1‐stearoyl‐2‐lignoceroyl‐sn‐glycerol, was synthesized as a chiral 1,2‐diacyl‐sn‐glycerol. The synthesis followed a previously reported procedure [41], with stearoyl and lignoceroyl chains introduced at the sn‐1 and sn‐2 positions, respectively. The synthesized compound was then purified using silica gel chromatography for further analysis and experimentation.

SLRI is an integrated workflow built upon standard confocal Raman microscopy combined with structured spectral acquisition and downstream spectral unmixing analysis. Specifically, Raman hyperspectral data are acquired in a point‐scanning manner using a confocal Raman microscope, providing spatially resolved spectral information at micrometer resolution. For 3D Raman imaging, sequential Z‐stack scanning is performed to generate volumetric hyperspectral datasets, which are reconstructed according to their original spatial coordinates. Following data acquisition, spectral preprocessing, reference‐based chemometric unmixing using non‐negative least squares (NNLS), and spatial reconstruction are performed. This method helped to separate the intricate Raman signals into distinct component biomolecular signatures. NNLS‐derived molecular maps represent relative abundance distributions constrained by the reference spectral library, rather than absolute molecular concentration maps. The unmixed spectra obtained were then utilized to generate 2D spatial distribution maps for each biomolecule [42, 43, 44]. Subsequently, the 3D reconstruction was conducted based on the 2D Raman datasets, allowing for a more comprehensive visualization and analysis of the spatial distribution of the biomolecules within the testicular tissues.

All Raman measurements were conducted using a SuperVision Medicine Bio‐SV confocal Raman microscope [45]. The microscope was equipped with a 532 nm solid‐state excitation laser, a 63× water‐immersion objective lens (Zeiss W Plan Apochromat 63×, N.A = 1), a 10 µm diameter multimode fiber‐optic cable, and a thermoelectrically cooled electron‐multiplying charge‐coupled device (EMCCD) detector. During the measurements, the laser power was maintained at 20 mW, and each spectrum was acquired with an integration time of 0.1 s. Acquisition parameters for 2D and 3D Raman imaging datasets were presented in Table S2. Raman maps were acquired over a spectral range of 300–3 800 cm^−^^1^. The 3D reconstructions were primarily used to visualize spatial distributions within defined regions of interest. For 3D imaging, the testicualr sample was continuously scanned along the Z‐axis to acquire Z‐stack datasets. The X‐Y step size was 1 µm, and the Z‐stacks step size ranged from 2 to 5.6 µm. Variations in Z‐axis sampling parameters and the number of optical sections mainly arise from differences in the physical thickness of the samples. The 2D molecular distribution maps obtained from consecutive Z‐planes were sequentially stacked according to the original XYZ coordinates recorded by the microscope. 3D reconstruction and visualization were performed using Java SE Development Kit 25.0.3. Because all Z‐stack datasets were acquired continuously within the same field of view and under the same microscope coordinate system, no additional cross‐correlation‐based intensity registration or lateral shift correction was applied.

### 5.8 Statistics and Reproducibility

Data were presented as mean ± standard error of the mean (SEM. Statistical analysis was performed using GraphPad Prism software Version 6.01 (GraphPad Inc., La Jolla, CA, USA). For the functional analysis, parametric comparisons were performed using either one‐way ANOVA for multiple groups or a Student's *t*‐test for two groups. For the SLRI analysis, the relative concentrations obtained via spectral unmixing/fitting against Raman reference spectra were displayed in violin plots and compared using a Student's *t*‐test. *P* < 0.05 was considered statistically significant.

## Author Contributions

JY, BZ, ZS, XZ, XT, XC, and CQ initiated the project, designed the study, coordinated the experiment, and wrote the manuscript. JL, XC, YQ, CQ, QH, DZ, LD, WQ, YS, XH, YS, KW, Z‐T‐Y, HZ, YD, AD, JF, JC, and YZ performed the experiments, analyzed the data, and provided conceptual inputs for the paper. All authors read and approved the final manuscript.

## Conflicts of Interest

The authors declare no competing interests.

## Supporting information




**Supporting File 1**: advs76757‐sup‐0001‐SuppMat.docx.

**Supporting File 2**: advs76757‐sup‐0002‐MovieS1.mp4.

**Supporting File 3**: advs76757‐sup‐0003‐MovieS2.mp4.

**Supporting File 4**: advs76757‐sup‐0004‐MovieS3.mp4.

**Supporting File 5**: advs76757‐sup‐0005‐MovieS4.mp4.

**Supporting File 6**: advs76757‐sup‐0006‐MovieS5.mp4.

**Supporting File 7**: advs76757‐sup‐0007‐MovieS6.mp4.

## Data Availability

The data are available from the corresponding author upon reasonable request.
