# Supplementary material for: Super‐Multiplexed Label‐Free Raman Imaging Uncovers Novel Testicular Metabolic Couplings for Residual Body and Spermatogonial Differentiation
Source: Adv Sci (Weinh). 2026 Jul 21;13(58):e76757. doi: 10.1002/advs.76757 (PMC13387037; doi:10.1002/advs.76757)
Supplement: Supplementary file 1 [file ADVS-13-e76757-s003.docx]

**Supplementary Materials**

**Supplementary Figure 1. Phenotypic analysis in testes.** (A) Co-staining of Orb2 and dpERK in testes. (B) Co-staining of Orb and dpERK in testes. (C) Intensity of UFA in Stage 1 and Stage 2 testes. (D) Intensity of SFA in Stage 1 and Stage 2 testes. (E) Intensity of Cyt C (ox) in Stage 1 and Stage 2 testes. DNA was stained with Hoechst33342. P<0.05 was considered statistically significant. Scale bar: 50 μm.

**Supplementary Figure 2. Knockdown of *Cyp4ae1* results in spermatogonia differentiation defects in testes.** (A) Interference efficiency verification in control and *Bam>Cyp4ae1 RNAi* testes. (B) Interference efficiency verification in control and *Bam>Cyp4ae1 RNAi-II* (the other RNAi target) testes. (C) Immunostaining of Vasa and 1B1 in control, *Bam>Cyp4ae1 RNAi-II* and *Bam>Cyp4ae1 RNAi-II; Δ86/+* testes. (D) The number of 1B1-positive punctate fusomes. (E) Immunostaining of PH3 and FasIII in control, *Bam>Cyp4ae1 RNAi-II* and *Bam>Cyp4ae1 RNAi-II; Δ86/+* testes. (F) The number of PH3-positive cells. (G) Staining of LD540 in control, *Bam>Cyp4ae1 RNAi-II* and *Bam>Cyp4ae1 RNAi-II; Δ86/+* testes. (H) The number of LD540-positive cells. DNA was stained with Hoechst33342. P<0.05 was considered statistically significant. Scale bar: 50 μm.

**Supplementary Figure 3. Detection of mitochondrial related indicators for *Cyp4ae1* in testes.** (A) The ratio of NADP+/NADPH in control, *Bam>Cyp4ae1 RNAi* and *Bam>Cyp4ae1 RNAi; Δ86/+* testes. (B) The ATP levels in control, *Bam>Cyp4ae1 RNAi* and *Bam>Cyp4ae1 RNAi; Δ86/+* testes. P<0.05 was considered statistically significant.

**Supplementary Figure 4. Spectral features between cytodynamic RBs and arrested spermatogonial clusters.** (A-B) Substance comparison for regions of detached RBs (II), attached RBs (IV), and early, mid, late-stages of arrested spermatogonial clusters (VII, VIII, IX). (C) Average spectrum features (300-3800 cm⁻¹) from selected regions of cytodynamic RBs and arrested spermatogonial clusters. (D-E) Average spectrum characteristics of specific segments for 300-1800 cm⁻¹ (D) and 2800-3100 cm⁻¹ (E) from selected regions of cytodynamic RBs and arrested spermatogonial clusters. Scale bar: 50 μm.

**Supplementary Figure 5. Substances for oxidative stress in cytoplasmic RBs and arrested spermatogonia.** (A) 2D/3D distributions and Raman spectra of GSSG in testes. (B) 2D/3D distributions and Raman spectra of GSH in testes. (C) 2D/3D distributions and Raman spectra of SOD in testes. (D) 2D/3D distributions and Raman spectra of 8-OHdG in testes. (E) 2D/3D distributions and Raman spectra of 8-iso PGF2α in testes. (F) 2D/3D distributions and Raman spectra of S-LG in testes. The color shown at the right end of the colorbar represents relatively higher signal intensity. Scale bar: 50 μm.

**Supplementary Figure 6. Substances for glucose metabolism in cytoplasmic RBs and arrested spermatogonia.** (A) 3D distributions and Raman spectra of Fru in testes. (B) 3D distributions and Raman spectra of Pyr in testes. The color shown at the top of the colorbar represents relatively higher signal intensity. Scale bar: 50 μm.

**Supplementary Figure 7. Non-targeted metabolomics analysis in control, *Bam>Cyp4ae1 RNAi* and *Bam>Cyp4ae1 RNAi; Δ86/+* testes.** (A) The number of differential metabolites. (B) A heatmap visualization of representative differential metabolites within the lipids and lipid-like molecules class, comparing control and *Bam>Cyp4ae1 RNAi* testes. (C) A heatmap visualization of representative differential metabolites within the lipids and lipid-like molecules class, comparing control and *Bam>Cyp4ae1 RNAi; Δ86/+* testes.

**Supplementary Figure 8. Specific distribution patterns for lipid subtypes in cytoplasmic RBs.** (A) Detailed distribution patterns of Raman imaging for neutral lipids (TAG Mix, DAG 18:0/24:0, Chol and CE) in cytoplasmic RBs. (B) Detailed distribution pattern of Raman imaging for phospholipid (PC) in cytoplasmic RBs. (C) Detailed distribution patterns of Raman imaging for fatty acids (TPA, PA and Ara) in cytoplasmic RBs. The color shown at the right end of the colorbar represents relatively higher signal intensity. Scale bar: 10 μm.

**Supplementary Figure 9. Spectral features of specific loci in cytodynamic RBs.** (A) Selected loci of punctate particles (a, b, c) at the periphery of the cytoplasmic RBs. (B) Selected diffuse loci (d, e, f) at the center of the cytoplasmic RBs. (C) Substance comparison for selected loci (a-f) from cytodynamic RBs. (D-E) Spectrum characteristics of specific segments for 300-1800 cm⁻¹ (D) and 2800-3100 cm⁻¹ (E) from selected loci of cytodynamic RBs. Scale bar: 10 μm.

**Supplementary Figure 10. Analysis of differential metabolites for amino acid metabolism in control, *Bam>Cyp4ae1 RNAi* and *Bam>Cyp4ae1 RNAi; Δ86/+* testes.** (A) A heatmap visualization of representative differential metabolites for amino acid metabolism comparing control and *Bam>Cyp4ae1 RNAi* testes. (B) A heatmap visualization of representative differential metabolites for amino acid metabolism comparing control and *Bam>Cyp4ae1 RNAi; Δ86/+* testes.

**Supplementary Figure 11. Specific distribution patterns for amino acids and vitamins in arrested spermatogonial clusters.** (A-F) Separate Raman images from 3D data of testicular apex for Arg (A), Met (B), Cit (C), Vit B6 (D), Vit B1 (E) and Vit C (F). The color shown at the top of the colorbar represents relatively higher signal intensity. Scale bar: 50 μm.

**Supplementary Figure 12. Specific distribution patterns for amino acids and vitamins in cytoplasmic RBs.** (A) Details for Raman imaging of Arg, Thr, Met and Cit in cytoplasmic RBs. (B) Details for Raman imaging of Vit B6, Vit B1, Vit C and Vit E in cytoplasmic RBs. The color shown at the right end of the colorbar represents relatively higher signal intensity. Scale bar:10 μm.

**Supplementary Table 1. Raman spectral library used in this study.**

| **Substance Name** | **Source** | **Catalog Number** |
| --- | --- | --- |
| SFA | Sigma‑Aldrich | S4751 |
| UFA | Sigma‑Aldrich | L1376 |
| Cyt C (ox) | Sigma‑Aldrich | C7752 |
| NADP^+^ | TargetMol | T4448 |
| NADPH | Beyotime | ST360 |
| ATP | MCE | HY-B2176R |
| TAG (mix) | Sigma‑Aldrich | 17810 |
| DAG (18:0/24:0) | NA | NA |
| Chol | Sigma-Aldrich | C8667 |
| CE | Merck | 700186M |
| PC | Sigma-Aldrich | P7443 |
| DSPI | AvantiResearch | 850143 |
| PA | MCE | HY-N0830R |
| TPA | Cayman Chemical | 10005157 |
| Ara | MCE | HY-W004260 |
| AA | Merck | 181198 |
| Acetyl-CoA | Yuanye Bio-Technology | S35407 |
| CIT | Yuanye Bio-Technology | S11150 |
| α-KG | Sigma-Aldrich | K1128 |
| Succ | Yuanye Bio-Technology | S30140 |
| G-6-P | Macklin | D729260 |
| F-6-P | TargetMol | T37985 |
| 3-PG | Macklin | D912712 |
| PEP | Solarbio life sciences | P8800 |
| Fru | Yuanye Bio-Technology | V30113 |
| Pyr | Merck | 113-24-6 |
| GSSG | MCE | HY-D0844 |
| GSH | MCE | HY-D0187 |
| SOD | MCE | HY-129064 |
| 8-OHdG | MCE | HY-W011540 |
| 8-iso PGF2α | Cayman Chemical | 16350 |
| S-LG | Sigma-Aldrich | L7140 |
| Arg | Sangon Biotech | A610206 |
| Thr | Sangon Biotech | A610919 |
| Val | Sangon Biotech | A600172 |
| Met | Sangon Biotech | A610346 |
| Ser | Sangon Biotech | A601479 |
| Cit | Yuanye Bio-Technology | S20014 |
| Vit B6 | Sangon Biotech | A600797 |
| Vit B1 | Sangon Biotech | A500986 |
| Vit C | Sangon Biotech | A610021 |
| Vit E | Sangon Biotech | A506771 |

**Supplementary Table 2. Acquisition parameters for 2D and 3D Raman imaging datasets**

| **Sample** | **2D** | | **3D** | | | | |
| --- | --- | --- | --- | --- | --- | --- | --- |
|  | **Image size (X × Y pixels)** | **X-Y step size**  **(μm)** | **Image size (X × Y pixels)** | **X-Y step size (μm)** | **Z-stacks step size (μm)** | **Number of Z-planes** | **Total imaging depth (μm)** |
| **Stage1** | 600*686 | 1 | 263*117 | 1 | 5.6 | 9 | 50.4 |
| **Stage2** | 541*649 | 1 | 149*273 | 1 | 2 | 20 | 40 |
| **Bam>Cyp4ae1 RNAi** | 671*522 | 1 | 126*249 | 1 | 4 | 9 | 36 |
| **Bam>Cyp4ae1 RNAi; Δ86/+** | 829*566 | 1 | 485*130 | 1 | 4 | 9 | 36 |
